# Sustained Low-Level Transmission of Zika and Chikungunya Viruses after Emergence in the Fiji Islands

**DOI:** 10.3201/eid2508.180524

**Published:** 2019-08

**Authors:** Mike Kama, Maite Aubry, Taina Naivalu, Jessica Vanhomwegen, Teheipuaura Mariteragi-Helle, Anita Teissier, Tuterarii Paoaafaite, Stéphane Hué, Martin L. Hibberd, Jean-Claude Manuguerra, Ketan Christi, Conall H. Watson, Eric J. Nilles, John Aaskov, Colleen L. Lau, Didier Musso, Adam J. Kucharski, Van-Mai Cao-Lormeau

**Affiliations:** Fiji Centre for Communicable Disease Control, Suva, Fiji (M. Kama);; The University of the South Pacific, Suva (M. Kama, T. Naivalu, K. Christi);; Institut Louis Malardé, Papeete, Tahiti (M. Aubry, T. Mariteragi-Helle, A. Teissier, T. Paoaafaite, D. Musso, V.-M. Cao-Lormeau);; Fiji National University, Suva (T. Naivalu);; Institut Pasteur, Paris, France (J. Vanhomwegen, J.-C. Manuguerra);; London School of Hygiene and Tropical Medicine, London, UK (S. Hué, M.L. Hibberd, C.H. Watson, A.J. Kucharski);; World Health Organization, Suva (E.J. Nilles);; Harvard Medical School and Brigham and Women’s Hospital, Boston, Massachusetts, USA (E.J. Nilles);; Harvard Humanitarian Initiative, Cambridge, Massachusetts, USA (E.J. Nilles);; Queensland University of Technology, Brisbane, Queensland, Australia (J. Aaskov);; Australian National University, Canberra, Australian Capital Territory, Australia (C.L. Lau);; Aix Marseille University, Marseille, France (D. Musso)

**Keywords:** Zika, chikungunya, dengue, Fiji, Pacific, seroprevalence, phylogeny, surveillance, arboviruses, mosquitoborne diseases, viruses, vector-borne infections

## Abstract

Zika and chikungunya viruses were first detected in Fiji in 2015. Examining surveillance and phylogenetic and serologic data, we found evidence of low-level transmission of Zika and chikungunya viruses during 2013–2017, in contrast to the major outbreaks caused by closely related virus strains in other Pacific Island countries.

Zika virus and chikungunya virus (CHIKV) have caused outbreaks in several tropical regions, including the Pacific (*1*). The first known Zika virus outbreak occurred in Yap Island (Federated States of Micronesia) in 2007 (*2*), followed by an explosive outbreak in French Polynesia in 2013–2014 (*3*), then other Pacific islands (*4*) and Latin America (*5*). CHIKV first appeared in the Pacific in 2011 (*6*), causing multiple outbreaks from 2013 onward (*4*).

In Fiji, the first confirmed Zika virus infections were detected in July 2015; these were locally acquired. By March 2016, a total of 13 confirmed infections had been reported (*7*). The first recorded CHIKV infection was an imported case detected in March 2015 (*8*); 24 autochthonous infections were identified by June 2016 (*9*). CHIKV and Zika virus were subsequently detected in travelers returning from Fiji (*10*,*11*). Outbreaks of dengue virus (DENV) have been recorded in Fiji (*4*,*12*), and evidence from other settings indicates that DENV and Zika virus can exhibit similar transmission characteristics in the same location (*13*). Despite enhanced surveillance, no large outbreaks of Zika or chikungunya were identified in Fiji, unlike in other settings (*3*,*4*). We describe the introduction, epidemiology, and transmission of Zika virus and CHIKV in Fiji during 2013–2017, in a context of concurrent circulation of DENV (*4*,*12*).

## The Study

We retrieved surveillance data for patients with prolonged fever (PF), defined as any fever lasting >3 days, and acute fever and rash (AFR) in Fiji (Figure), as well as data on suspected and confirmed Zika virus, CHIKV, and DENV infections (Appendix Table 1). We reconstructed phylogenetic trees of Zika virus and CHIKV sequences by using Bayesian inference (Appendix Tables 2, 3). We recruited 778 participants in Fiji during September–November 2013 as part of a community-based serologic survey (Appendix Figure 1). We collected follow-up samples from the same participants in the Central Division (N = 333) during October–November 2015. We tested serum samples by using a recombinant antigen-based microsphere immunoassay to detect Zika virus, CHIKV, and DENV-1–4 IgG (Appendix). Analysis of neutralizing antibodies against Zika virus and DENV in a subset of 69 paired serum samples showed good concordance with the microsphere immunoassay for Zika virus (κ = 0.71) and DENV (κ = 0.80) (Appendix Table 4).

**Figure:**
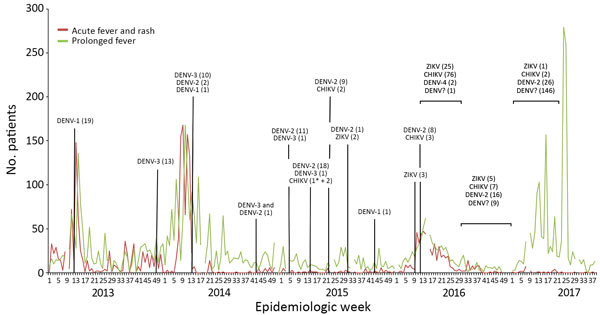
Reports of patients with acute fever and rash, prolonged fever, and infections with dengue, Zika, or chikungunya viruses confirmed by reverse transcription PCR in Fiji, 2013–2017. Number of dengue, Zika, or chikungunya virus infections were confirmed by reverse transcription PCR. Asterisks (*) indicate imported chikungunya virus infections. CHIKV, chikungunya virus; DENV-1, dengue virus serotype 1; DENV-2, dengue virus serotype 2; DENV-3, dengue virus serotype 3; DENV-4, dengue virus serotype 4; DENV?, information on dengue virus serotype not available; ZIKV, Zika virus.

Surveillance data recorded during 2013–2017 indicated cyclical increases in AFR and PF each year, concurrent with the hot and rainy season occurring in December–April (Figure). Molecular testing of blood samples from symptomatic patients suggested outbreaks of DENV-1 in 2013, DENV-3 in 2014, and DENV-2 in 2017. In 2015, Zika virus and CHIKV apparently were co-circulating at low levels alongside DENV-1, DENV-2, and DENV-3. In 2016, an increase in proportional positivity for Zika virus and CHIKV was detected among 804 AFR and PF patients, suggesting higher transmission of these viruses but not widespread circulation (Zika virus, 32/804 [4%]; CHIKV, 86/804 [11%]; DENV-2, 10/804 [1%]; DENV-4, 2/804 [<1%]). Additional CHIKV (n = 2) and Zika virus (n = 1) infections were detected during the first half of 2017.

We aligned the envelope (E) gene sequences of Zika virus strains collected in Fiji during 2015–2016 (Appendix Table 2) with sequences from other countries. All Zika virus strains belonged to the Asia lineage and segregated into 2 separate clades (posterior probability >0.99) (Appendix Figure 2, panel A). The Fiji Zika virus strains belonged to the Asia and Oceania clade; 2 strains collected in 2016 grouped with viruses isolated in Japan in 2016 (posterior probability >0.99), including 1 from a traveler returning from Fiji. The estimated time of most recent common ancestor of this cluster was September 2013 (95% higher probability density [HPD] interval September 2011–August 2015). The remaining Fiji strains formed a distinct cluster with strains from Southeast Asia and other Pacific Islands. We dated the origin of this second cluster to November 2013 (95% HPD interval March 2013–July 2015). 

We aligned the E1 gene sequences of Fiji CHIKV strains collected during 2015–2016 (Appendix Table 2) with sequences from other countries. All strains belonged to the Asia genotype; Fiji strains formed a monophyletic group with strains from Tonga sampled in 2014 (posterior probability 1.00) (Appendix Figure 2, panel B). This grouping suggested a single introduction of CHIKV into Fiji in February 2014 (95% HPD interval December 2013–August 2014) and subsequent persistence in the population.

Zika virus seroprevalence in 2013 was 7.8% (95% CI 6.1%–10%); we observed no significant differences between age groups, sexes, residential divisions, or areas (Table). In 2015, seroprevalence was 21.9% (95% CI 17.6%–26.8%), and the only significant difference observed was between rural (14.2% [95% CI 8.3%–22%]) and urban (26.6% [95% CI 19.5%–34.6%]) areas (p = 0.0202). Compared with 2013, Zika virus seroprevalence in 2015 was significantly higher overall (p<0.0001). However, no change was observed in the CHIKV seroprevalence between 2013 (0.8% [95% CI 0.3%–1.7%]) and 2015 (0.9% [95% CI 0.2%–2.6%]), and no significant differences were observed in the demographic variables described for Zika virus. The seroprevalence of DENV in 2013 was 73% (95% CI 69.7%–76.1%) and was lower among persons in the 0–19 years age group compared with other age groups (p<0.0001) (Table). We observed no significant difference by sex. DENV seropositivity was higher in the Northern than in the Central and Western divisions (p<0.0368) and higher in urban than in rural areas (p = 0.0136). During 2013–2015, we observed a significant increase in DENV seroprevalence (82.9% [95% CI 78.4%–86.8%]; p = 0.0004) among persons 0–19 years of age (p = 0.0013), women and girls (p = 0.0002), and participants living in the Central Division (p = 0.0018) and urban areas (p = 0.0048). Seroprevalence in 2015 remained lower in persons 0–19 years of age than in other age groups (p<0.0137) but was significantly higher in women and girls compared with men and boys (p = 0.039) and in urban compared with rural areas (p = 0.0053).

**Table:** Prevalence of Zika, chikungunya, and dengue virus antibodies in a representative subset of the population sampled during September–November 2013 and October–November 2015, Fiji Islands*

Variable	No. seropositive/no. tested (% [95% CI])							
	Zika virus			Chikungunya virus			Dengue viruses†	
	2013	2015		2013	2015		2013	2015
Total	61/778‡ (7.8 [6.1–10])	73/333 (21.9 [17.6–26.8])		6/778 (0.8 [0.3–1.7])	3/333 (0.9 [0.2–2.6])		568/778 (73 [69.7–76.1])	276/333 (82.9 [78.4–86.8])
Age range (median), y	2–85 (28)	4–80 (29)		2–85 (28)	4–80 (29)		2–78 (28)	4–80 (29)
Age group, y								
0–19	29/282 (10.3 [7–14.4])	29/115 (25.2 [17.6–34.2])		4/282 (1.4 [0.4–3.6])	1/115 (0.9 [0–4.7])		141/282 (50 [44–56])	78/115 (67.8 [58.5–76.2])
20–39	15/239 (6.3 [3.6–10.1])	18/103 (17.5 [10.7–26.2])		1/239 (0.4 [0–2.3])	1/103 (1 [0–5.3])		201/239 (84.1 [78.8–88.5])	93/103 (90.3 [82.9–95.2])
40–59	11/179 (6.1 [3.1–10.7])	13/73 (17.8 [9.8–28.5])		1/179 (0.6 [0–3.1])	1/73 (1.4 [0–7.4])		161/179 (89.9 [84.6–93.9])	68/73 (93.2 [84.7–97.7])
≥60	6/77 (7.8 [2.9–16.2])	13/42 (31 [17.6–47.1])		0/77 (0 [0–4.7])	0/42 (0 [0–8.4])		64/77 (83.1 [72.9–90.7])	37/42 (88.1 [74.4–96])
Sex								
F	28/423 (6.6 [4.4–9.4])	41/190 (21.6 [16–28.1])		4/423 (0.9 [0.3–2.4])	2/190 (1.1 [0.1–3.8])		312/423 (73.8 [69.3–77.9])	165/190 (86.8 [81.2–91.3])
M	33/354 (9.3 [6.5–12.8])	32/143 (22.4 [15.8–30.1])		2/354 (0.6 [0.1–2])	1/143 (0.7 [0–3.8])		255/354 (72 [67–76.6])	111/143 (77.6 [69.9–84.2])
Division								
Central	30/451 (6.7 [4.5–9.4])	73/333 (21.9 [17.6–26.8])		5/451 (1.1 [0.4–2.6])	3/333 (0.9 [0.2–2.6])		331/451 (73.4 [69.1–77.4])	276/333 (82.9 [78.4–86.8])
Northern	7/59 (11.9 [4.9–22.9])	ND		0/59 (0 [0–6.1])	ND		51/59 (86.4 [75–94])	ND
Western	24/268 (9 [5.8–13])	ND		1/268 (0.4 [0–2.1])	ND		186/268 (69.4 [63.5–74.9])	ND
Area								
Periurban	10/135 (7.4 [3.6–13.2])	19/77 (24.7 [15.6–35.8])		2/135 (1.5 [0.2–5.2])	0/77 (0 [0–4.7])		104/135 (77 [69–83.8])	66/77 (85.7 [75.9–92.6])
Rural	24/344 (7 [4.5–10.2])	16/113 (14.2 [8.3–22])		2/344 (0.6 [0.1–2.1])	0/113 (0 [0–3.2])		234/344 (68 [62.8–72.9])	84/113 (74.3 [65.3–82.1])
Urban	27/298 (9.1 [6.1–12.9])	38/143 (26.6 [19.5–34.6])		2/298 (0.7 [0.1–2.4])	3/143 (2.1 [0.4–6])		229/298 (76.8 [71.6–81.5])	126/143 (88.1 [81.6–92.9])

*No participants were recruited in the Northern and Western divisions in 2015. ND, no data. †Seropositivity for >1 serotypes of dengue virus. ‡For 1 participant, demographic data were not available except for the administrative division of residence.

Analysis of paired samples collected in 2013 and 2015 from the same participants supported previous serologic findings on all samples collected (Appendix Table 5). Among these participants, 55/311 (17.7% [95% CI 13.6%–22.4%]) seroconverted to Zika virus, 40/311 (12.9% [95% CI 9.3%–17.1%]) seroconverted to DENV, and 1/311 (0.3% [95% CI 0.008%–1.8%]) seroconverted to CHIKV (Appendix Table 6).

## Conclusions

We found evidence of low-level transmission of Zika virus and CHIKV in Fiji for multiple years after their initial introduction into a population that probably was immunologically naive, despite an ecologic environment subject to large and recurrent DENV outbreaks. Similar evidence of low-level Zika virus circulation has been observed in other settings (*14*). Our findings indicate that Zika virus circulated before the first confirmed cases in 2015 and that multiple introductions from other Pacific islands might have occurred, which suggests the possible role of Zika virus in a cluster of Guillain-Barré syndrome cases of unknown etiology in Fiji during February–May 2014 (*15*). However, there was no epidemiologic or serologic evidence that CHIKV circulated in Fiji before it was first reported in 2015. High DENV seroprevalence in 2013 and 2015 suggests that DENV is endemic in Fiji, with seroprevalence increasing with age. Our data also suggest that DENV and Zika virus transmission occurs mostly in urban areas where peridomestic mosquitoes, notably *Aedes aegypti* and *Ae. albopictus*, are abundant.

Our study highlights the difficulties in detecting and anticipating outbreaks of Zika virus and CHIKV and the value of having multiple data sources available. Stronger clinical and laboratory surveillance capacities are needed to ensure the early detection of these and future infectious disease threats.

Appendix Additional information for sustained low-level transmission of Zika and chikungunya viruses after emergence in the Fiji Islands.
