# Supplementary material for: Sustained Low-Level Transmission of Zika and Chikungunya Viruses after Emergence in the Fiji Islands
Source: Emerg Infect Dis. 2019 Aug;25(8):1535–8. doi: 10.3201/eid2508.180524 (PMC6649350; doi:10.3201/eid2508.180524)
Supplement: Appendix — Additional information for sustained low-level transmission of Zika and chikungunya viruses after emergence in the Fiji Islands. [file 18-0524-Techapp-s1.pdf]

Article DOI: https://doi.org/10.3201/eid2508.180524

Sustained Low-Level Transmission of Zika and Chikungunya Viruses after Emergence in the Fiji Islands

Appendix

Materials and Methods

Study Location and Population

The Republic of the Fiji islands is located in the South Pacific and comprises 322 islands divided into 4 administrative Divisions: Central, Western, Northern and Eastern (Appendix Figure 1). The population was 837,271 in 2007 and most of the inhabitants lived in the Central (41%), Western (38%) and Northern (16%) Divisions. The proportion of people living in urban/peri-urban and rural areas was similar (51% versus 49%). The median age of the population was 21 years with a 1:1 ratio of males to females.

Syndromic and Laboratory Surveillance

Surveillance data for patients with prolonged fever (PF, ie any fever lasting three or more days) and acute fever and rash (AFR) in Fiji (week 1 of 2013 to week 37 of 2017) were retrieved from the Pacific Syndromic Surveillance System weekly reports (*1*); the case definitions have been previously reported (*2*).

Data on suspected and confirmed DENV, CHIKV and ZIKV cases including type and location of testing were retrieved from the laboratory surveillance database of the Fiji Centre for Communicable Disease Control (FCCDC, Suva, Fiji) (Appendix Table 1). Molecular assays for DENV, CHIKV and ZIKV were primarily performed at the Institut Louis Malardé (ILM, Tahiti, French Polynesia). From January 2013, serum samples blotted on filter paper cards and saliva samples collected on dry oral swabs from patients with PF or AFR were referred to ILM for testing by real-time RT-PCR for the presence of RNA from the four DENV serotypes, CHIKV and ZIKV following the protocols previously described (*3*–*6*). From 2016, molecular testing for DENV, ZIKV and CHIKV was implemented at the FCCDC Laboratory (Mataika House) using

the QIAamp Viral RNA Mini Kit (Qiagen, Germany) and Trioplex Real-time RT-PCR assay provided by the Centers for Disease Control and Prevention (CDC, USA) (*7*). A smaller number of samples were tested using RT-PCR for the same pathogens at the Environmental Science and Research Limited (ESR, New Zealand) and the Environmental Health Institute (EHI, Singapore).

Sequencing of Viral Genes and Phylogenetic Analyses

The envelope (E) gene of ZIKV strains recovered from two saliva samples collected in 2015 and three serum samples collected in 2016, and the E1 gene of CHIKV strains isolated from two serum samples collected in 2015 and three additional serum samples collected in 2016 were sequenced (Appendix Table 2). Briefly, ZIKV and CHIKV RNA were extracted using the Easymag extraction system (bioMérieux, France) as previously detailed (4) and amplified using the One-Step RT-PCR Kit and HotstarTaq DNA Polymerase Kit or HotstarTaq Plus DNA Polymerase Kit (Qiagen, Germany) with primers described in Appendix Table 3. The PCR products were purified using the QIAquick PCR Purification Kit or the QIAquick Gel Extraction Kit (Qiagen, Germany). The subsequent sequencing reactions were performed with the BigDye® Terminator v3.1 Cycle Sequencing Kit (Applied Biosystems, USA) and the products were purified with the DyeEx 96 Kit (Qiagen, Germany). Sequencing of purified cDNA was performed on the 3500 Series Genetic Analyzer (Applied Biosystems, USA). Fragments of nucleotide sequences obtained for each ZIKV and CHIKV strain were cleaned and assembled using the Sequencher 4.10.1 software (Gene Codes Corporation, USA). Complete sequences of ZIKV E gene and CHIKV E1 gene were aligned with sequences retrieved from GenBank using the Clustal W algorithm integrated in the MEGA 7 phylogenetic package (*8*). The sequences retrieved from GenBank were selected using nucleotide BLAST searches (*9*), where all sequences with a reported date of sampling and country of origin sharing more than 99% genetic identity to the Fiji sequences were retained. After removal of duplicates, the ZIKV and CHIKV alignments contained 66 and 62 sequences, respectively. For each alignment, phylogenies in nucleotide substitutions per sites and in unit of time (‘dated’ phylogenies) were reconstructed by Bayesian MCMC inference, using the package BEAST (*10*).

Serosurveys

Sampling Design and Data Collection

Participants (N = 778) were recruited between September and November 2013 in the Northern, Western and Central divisions of Fiji (Appendix Figure 1), as part of a population-representative community-based typhoid/leptospirosis seroprevalence study as previously described (*11*,*12*). Briefly, local nursing zones were initially selected using population-proportionate sampling. Next, one community was randomly selected from each of these zones, then 25 households were selected from each community and finally one individual was selected from each of the households: household members (defined as someone who stayed at the house the previous night) were enumerated, with one selected at random. A questionnaire was used to collect demographic data (age, gender, Division, residential area), and a 5 ml venous blood sample was collected. A second phase to obtain paired samples was conducted in the Central Division between October and November 2015 (Appendix Figure 1). Field officers followed-up participants who had participated in the 2013 study, and who had consented to being contacted again for health research. Similar to 2013, a 5 ml venous blood sample was collected from the participants (N = 333) along with questionnaire data. This resulted in a set of paired samples (N = 311). Insufficient volumes of serum from some of the 2013 samples meant that a subset of participants (N = 22) only had 2015 samples available.

Informed Consent and Ethics Approvals

Ethics approvals were granted by the Fiji National Health Research Ethics Review Committee (FNRERC 2013–03, N°2015.114.NW and N°2015.45.MC), the University of the South Pacific (FSTER/2015/10/Research Proposal Approval/Mike Kama/S90058620 and FSTER/2015/11/Research Proposal Approval/Taina Naivalu/S11127279) and the London School of Hygiene & Tropical Medicine Observational Research Ethics Committee (6344 and 10207).

Serologic Analysis

Serologic assays to detect immunoglobulin class G (IgG) antibodies against ZIKV, CHIKV and the four serotypes of DENV were performed on serum samples collected in 2013 (N = 778) and 2015 (N = 333), including paired samples (N = 311), using a recombinant antigen-based microsphere immunoassay (MIA) as previously described (*13*–*15*), with recombinant antigens of ZIKV, CHIKV, DENV-1, –2, –3, or –4 (GenBank accession no. KJ776791,

AM258994, AF226686.1, FM986654, FJ44740.1, and FM986672.1 respectively). The presence of neutralizing antibodies against ZIKV and each of the four DENV serotypes was assessed in the paired serum samples of the 69 participants for whom at least one of the samples had anti-ZIKV antibodies detected by MIA, using a neutralization assay as previously detailed (*13*,*15*), with ZIKV, DENV-1, –2, –3 or –4 strains (GenBank accession no. KX369547, MG181997, JQ650020, AY744680, and KY933793, respectively). Among the samples reactive by MIA, 66/83 (79.5%) exhibited neutralizing activity for ZIKV ($\kappa = 0.71$) and 109/112 (97.3%) for DENV ($\kappa = 0.80$) (Appendix Table 4); showing good concordance between the MIA and neutralizing antibody assays for anti-ZIKV and anti-DENV IgG respectively.

Statistical Analysis

Statistical analysis of seroprevalence data was conducted with the GraphPad Prism 6 version 6.03 software using the Fisher's test. P values less than 0.05 were considered as statistically significant.

References

1. World Health Organization. Weekly Pacific Syndromic Surveillance Reports. [cited 2018 Jan 31]. http://www.wpro.who.int/southpacific/programmes/communicable_diseases/disease_surveillance_response/page/en/index2.html

2. Craig AT, Kama M, Samo M, Vaai S, Matanaicake J, Joshua C, et al. Early warning epidemic surveillance in the Pacific island nations: an evaluation of the Pacific syndromic surveillance system. Trop Med Int Health. 2016;21:917–27. PubMed http://dx.doi.org/10.1111/tmi.12711

3. Cao-Lormeau V-M, Roche C, Teissier A, Robin E, Berry A-L, Mallet H-P, et al. Zika virus, French Polynesia, South Pacific, 2013. Emerg Infect Dis. 2014;20:1085–6. PubMed http://dx.doi.org/10.3201/eid2011.141380

4. Aubry M, Roche C, Dupont-Rouzeyrol M, Aaskov J, Viallon J, Marfel M, et al. Use of serum and blood samples on filter paper to improve the surveillance of dengue in Pacific Island countries. J Clin Virol. 2012;55:23–9. PubMed http://dx.doi.org/10.1016/j.jcv.2012.05.010

5. Aubry M, Teissier A, Roche C, Richard V, Yan AS, Zisou K, et al. Chikungunya outbreak, French Polynesia, 2014. Emerg Infect Dis. 2015;21:724–6. PubMed http://dx.doi.org/10.3201/eid2104.141741

6. Musso D, Rouault E, Teissier A, Lanteri MC, Zisou K, Broult J, et al. Molecular detection of Zika virus in blood and RNA load determination during the French Polynesian outbreak. J Med Virol. 2017;89:1505–10. PubMed http://dx.doi.org/10.1002/jmv.24735

7. Centers for Disease Control and Prevention. Trioplex Real-time RT-PCR Assay [cited 2018 Jan 31]. https://www.cdc.gov/zika/pdfs/trioplex-real-time-rt-pcr-assay-instructions-for-use.pdf

8. Kumar S, Stecher G, Tamura K. MEGA7: Molecular Evolutionary Genetics Analysis version 7.0 for bigger datasets. Mol Biol Evol. 2016;33:1870–4. PubMed http://dx.doi.org/10.1093/molbev/msw054

9. Altschul SF, Gish W, Miller W, Myers EW, Lipman DJ. Basic local alignment search tool. J Mol Biol. 1990;215:403–10. PubMed http://dx.doi.org/10.1016/S0022-2836(05)80360-2

10. Drummond AJ, Suchard MA, Xie D, Rambaut A. Bayesian phylogenetics with BEAUti and the BEAST 1.7. Mol Biol Evol. 2012;29:1969–73. PubMed http://dx.doi.org/10.1093/molbev/mss075

11. Lau CL, Watson CH, Lowry JH, David MC, Craig SB, Wynwood SJ, et al. Human leptospirosis infection in Fiji: an eco-epidemiological approach to identifying risk factors and environmental drivers for transmission. PLoS Negl Trop Dis. 2016;10:e0004405. PubMed http://dx.doi.org/10.1371/journal.pntd.0004405

12. Watson CH, Baker S, Lau CL, Rawalai K, Taufa M, Coriakula J, et al. A cross-sectional seroepidemiological survey of typhoid fever in Fiji. PLoS Negl Trop Dis. 2017;11:e0005786. PubMed http://dx.doi.org/10.1371/journal.pntd.0005786

13. Cao-Lormeau V-M, Blake A, Mons S, Lastère S, Roche C, Vanhomwegen J, et al. Guillain-Barré Syndrome outbreak associated with Zika virus infection in French Polynesia: a case-control study. Lancet. 2016;387:1531–9. PubMed http://dx.doi.org/10.1016/S0140-6736(16)00562-6

14. Aubry M, Teissier A, Huart M, Merceron S, Vanhomwegen J, Roche C, et al. Zika virus seroprevalence, French Polynesia, 2014–2015. Emerg Infect Dis. 2017;23:669–72. PubMed http://dx.doi.org/10.3201/eid2304.161549

15. Aubry M, Teissier A, Huart M, Merceron S, Vanhomwegen J, Mapotoeke M, et al. Seroprevalence of dengue and chikungunya virus antibodies, French Polynesia, 2014–2015. Emerg Infect Dis. 2018;24:558–61. PubMed http://dx.doi.org/10.3201/eid2403.171149

16. Deng Y-Q, Zhao H, Li X-F, Zhang N-N, Liu Z-Y, Jiang T, et al. Isolation, identification and genomic characterization of the Asian lineage Zika virus imported to China. Sci China Life Sci. 2016;59:428–30. PubMed http://dx.doi.org/10.1007/s11427-016-5043-4

17. Lanciotti RS, Kosoy OL, Laven JJ, Velez JO, Lambert AJ, Johnson AJ, et al. Genetic and serologic properties of Zika virus associated with an epidemic, Yap State, Micronesia, 2007. Emerg Infect Dis. 2008;14:1232–9. PubMed http://dx.doi.org/10.3201/eid1408.080287

18. Stapleford KA, Moratorio G, Henningsson R, Chen R, Matheus S, Enfissi A, et al. Whole-genome sequencing analysis from the chikungunya virus Caribbean outbreak reveals novel evolutionary genomic elements. PLoS Negl Trop Dis. 2016;10:e0004402. PubMed http://dx.doi.org/10.1371/journal.pntd.0004402

19. Parola P, de Lamballerie X, Jourdan J, Rovery C, Vaillant V, Minodier P, et al. Novel chikungunya virus variant in travelers returning from Indian Ocean islands. Emerg Infect Dis. 2006;12:1493–9. PubMed http://dx.doi.org/10.3201/eid1210.060610

20. Pastorino B, Bessaud M, Grandadam M, Murri S, Tolou HJ, Peyrefitte CN. Development of a TaqMan RT-PCR assay without RNA extraction step for the detection and quantification of African Chikungunya viruses. J Virol Methods. 2005;124:65–71. PubMed http://dx.doi.org/10.1016/j.jviromet.2004.11.002

Appendix Table 1. Diagnosis of dengue, chikungunya and Zika virus infections in Fiji by RT-PCR between 2013–2017*

Period	Laboratory	Number tested	Number positive		
			DENV	ZIKV	CHIKV
2013					
January–June	ILM	29	19	NT	NT
July–December	ILM	35	13	NT	NT
2014					
January–June	ILM	36	13	0	0
July–December	ILM	3	1	0	0
2015					
January–June	ILM	344	40	0	5
July–December	ILM	69	2	2	0
2016					
January–June	ILM	187	12	3	3
January–June	Mataika House	325	2	6	67
January–June	ESR	96	3	16	16
January–June	EHI	196	NT	7	NT
July–December	ILM	15	11	0	0
July–December	Mataika House	233	8	1	0
2017					
January–June	ILM	27	26	NT	0
January–June	Mataika House	572	146	1	2

*ZIKV, Zika virus; CHIKV, chikungunya virus; DENV, dengue viruses; ILM, Institut Louis Malardé (French Polynesia); ESR, Environmental Science and Research Limited (New Zealand); EHI, Environmental Health Institute (Singapore); NT, not tested.

Appendix Table 2. Characteristics of Zika and chikungunya virus strains sequenced in this study

Virus	Strain ID	Geographic origin	Collection date	Sample origin	GenBank accession number
Zika	Fiji2015–110715–17A	Suva (Central division)	11 July 2015	Saliva	MG216928
Zika	Fiji2015–110715–18A	Suva (Central division)	11 July 2015	Saliva	MG216929
Zika	Fiji2016–260516–7284	Lautoka (Western division)	26 May 2016	Serum	MG216930
Zika	Fiji2016–220716–1568	Suva (Central division)	22 July 2016	Serum	MG216931
Zika	Fiji2016–030816–1580	Lautoka (Western division)	03 August 2016	Serum	MG216932
Chikungunya	Fiji2015–070315–0515	Lautoka (Western division)	07 March 2015	Serum	MG271970
Chikungunya	Fiji2015–250315–0815	Nadi (Western division)	25 March 2015	Serum	MG271971
Chikungunya	Fiji2016–060516–5530	Suva (Central division)	06 May 2016	Serum	MG271972
Chikungunya	Fiji2016–070516–5541	Suva (Central division)	07 May 2016	Serum	MG271973
Chikungunya	Fiji2016–060517–5561	Suva (Central division)	06 May 2016	Serum	MG271974

Appendix Table 3. Primers used for sequencing of the envelope gene of Zika virus and envelope gene (E1) of chikungunya virus

Virus	Primer	Genome position	Sequence (5′→3′)	Reference
Zika	ZIKVF2	782–801	CGCAAACCTGGTTGGAATCA	(*16*)
Zika	ZIKV 835	882–904	TTGGTCATGATACTGCTGATTGC	(*17*)
Zika	ZIKV 911c	958–937	CCTTCCACAAAGTCCCTATTGC	(*17*)
Zika	ZIKV 1086	1133–1149	CCGCTGCCCAACACAAG	(*17*)
Zika	ZIKV 1162c	1209–1186	CCACTAACGTTCTTTTGCAGACAT	(*17*)
Zika	ZIKVF3	1510–1530	GGAAGCCTAGGACTTGATTGT	(*16*)
Zika	ZIKVR2	1729–1709	CCACGACAGTTTGCCTTTTGG	(*16*)
Zika	ZIKVF4	2172–2193	CAGCACCATTGGAAAAGCATTT	(*16*)
Zika	ZIKVR3	2487–2466	CGAGCACCCCACATCAGCAGAG	(*16*)
Zika	ZIKVR4	2952–2928	GAACCCATGATCCTCCACAAGAAAG	(*16*)
Chikungunya	CHIK17F	8976–8997	GGAACTACCTTGCAGCACGTAC	(*18*)
Chikungunya	CHIKE1S1	10118–10139	ACATCACGTGCGAGTACA	(*19*)
Chikungunya	CHIK19F	10172–10191	GTACAGCAGAGTGTAAGGACCA	(*18*)
Chikungunya	F-CHIK	10366–10387	AAGCTYCGCGTCCTTTACCAAG	(*20*)
Chikungunya	R-CHIK	10574–10554	CCAAATTGTCCYGGTCTTCCT	(*20*)
Chikungunya	CHIK 18R	10989–10969	GGCGTTAGTCATCGAGTGCAC	(*18*)
Chikungunya	CHIKE1R1	11491–11470	TCTCTTAAGGGRCACATATACC	(*19*)
Chikungunya	CHIK 21F	10803–10824	GAACATGCCTATCTCCATCGAC	(*18*)

Appendix Table 4. Comparison of the detection of anti-Zika or anti-dengue virus IgG by microsphere immunoassay and seroneutralization assay in 69 paired samples from participants collected during September-November 2013 and October-November 2015 in the Central division in Fiji*

Year (no. samples tested)	MIA / NTA							
	ZIKV + / +	ZIKV - / -	ZIKV + / -	ZIKV - / +	DENV + / +†	DENV - / -	DENV + / -	DENV - / +†
2013 (N = 69)	6	51	9	3	46	17	3	3
2015 (N = 69)	60	1	8	0	63	4	0	2
Total (N = 138)	66	52	17	3	109	21	3	5

*MIA, microsphere immunoassay; NTA, Neutralization assay; ZIKV, Zika virus; DENV, dengue viruses; +, positive serologic result; -, negative serologic result.
†Neutralization of one or more serotypes of dengue virus.

Appendix Table 5. Prevalence of anti-Zika, chikungunya or dengue virus antibodies in the paired samples (N = 311) collected during September-November 2013 and October-November 2015 in the Central division in Fiji*

	No. seropositive / No. tested (% [95% CI])					
	ZIKV		CHIKV		DENV†	
Variable	2013	2015	2013	2015	2013	2015
Total	16/311 (5.1 [3–8.2])	69/311 (22.2 [17.7–27.2])	3/311 (1 [0.2–2.8])	3/311 (1 [0.2–2.8])	228/311 (73.3 [68–78.1])	256/311 (82.3 [77.6–86.4])
Age range (median)	2–78 (28)	4–80 (29)	2–78 (28)	4–80 (29)	2–78 (28)	4–80 (29)
Age groups						
0–19	10/121 (8.3 [4–14.7])	25/105 (23.8 [16–33.1])	2/121 (1.7 [0.2–5.8])	1/105 (1 [0–5.2])	63/121 (52.1 [42.8–61.2])	69/105 (65.7 [55.8–74.7])
20–39	3/91 (3.3 [0.7–9.3])	18/97 (18.6 [11.4–27.7])	0/91 (0 [0–4])	1/97 (1 [0–5.6])	73/91 (80.2 [70.6–87.8])	85/97 (87.6 [79.4–93.4])
40–59	1/65 (1.5 [0–8.3])	14/72 (19.4 [11.1–30.5])	1/65 (1.5 [0–8.3])	1/72 (1.4 [0–7.5])	60/65 (92.3 [83–97.5])	67/72 (93.1 [84.5–97.7])
60+	2/34 (5.9 [0.7–19.7])	12/37 (32.4 [18–49.8])	0/34 (0 [0–10.3])	0/37 (0 [0–9.5])	32/34 (94.1 [80.3–99.3])	35/37 (94.6 [81.8–99.3])
Gender						
Female	7/177 (4 [1.6–8])	39/177 (22 [16.2–28.9])	12/177 (6.8 [3.6–11.5])	2/177 (1.1 [0.1–4])	133/177 (75.1 [68.1–81.3])	152/177 (85.9 [79.9–90.6])
Male	9/134 (6.7 [3.1–12.4])	30/134 (22.4 [15.6–30.4])	1/134 (0.7 [0–4.1])	1/134 (0.7 [0–4.1])	95/134 (70.9 [62.4–78.4])	104/134 (77.6 [69.6–84.4])
Zone						
Periurban	4/65 (6.2 [1.7–15])	18/65 (27.7 [17.3–40.2])	0/65 (0 [0–5.5])	0/65 (0 [0–5.5])	53/65 (81.5 [70–90.1])	54/65 (83.1 [71.7–91.2])
Rural	4/110 (3.6 [1–9])	16/110 (14.5 [8.5–22.5])	1/110 (0.9 [0–5])	0/110 (0 [0–3.3])	70/110 (63.6 [53.9–72.6])	83/110 (75.5 [66.3–83.2])
Urban	8/136 (5.9 [2.6–11.3])	35/136 (25.7 [18.6–33.9])	2/136 (1.5 [0.2–5.2])	3/136 (2.2 [0.5–6.3])	105/136 (77.2 [69.2–84])	119/136 (87.5 [80.7–92.5])

*ZIKV, Zika virus; CHIKV, chikungunya virus; DENV, dengue viruses.
†Seropositivity for one or more serotypes of dengue virus.

Appendix Table 6. Detection of IgG against Zika, chikungunya and dengue viruses detected by microsphere immunoassay in the paired samples from participants (N = 311) collected during September-November 2013 and October-November 2015 in the Central division in Fiji*.

	2015					
2013	ZIKV+	ZIKV-	CHIKV+	CHIKV-	DENV+†	DENV-
ZIKV+	14	2	–	–	–	–
ZIKV-	55	240	–	–	–	–
DENV+†	–	–	–	–	216	12
DENV-	–	–	–	–	40	43
CHIKV+	–	–	2	1	–	–
CHIKV-	–	–	1	307	–	–

*ZIKV, Zika virus; CHIKV, Chikungunya virus; DENV, dengue viruses.
†Seropositivity for one or more serotypes of dengue virus.

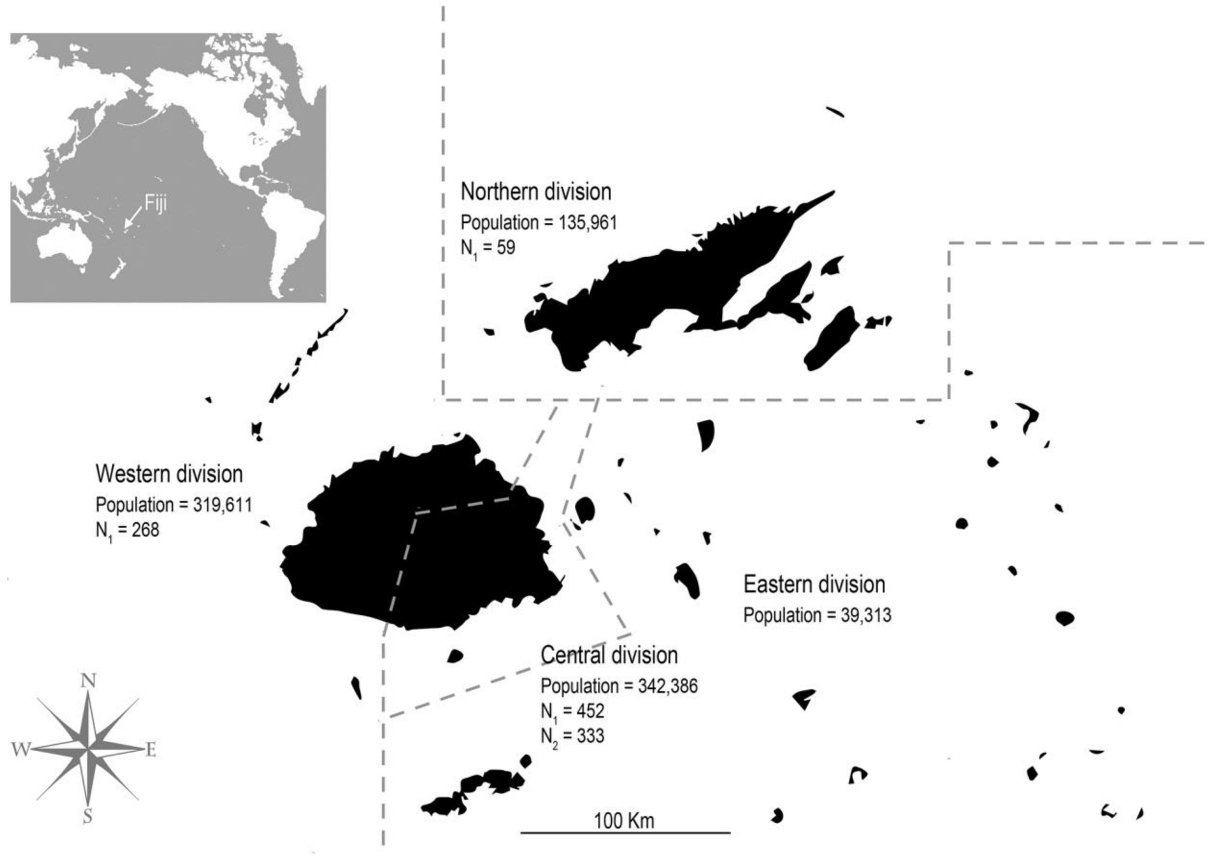

Appendix Figure 1. Geographic distribution of participants recruited among the general population of Fiji between September-November 2013 and October-November 2015. N1 and N2 indicate areas of recruitment in 2013 and 2015, respectively. The total population for each area is given. Gray dashed lines delineate the 4 divisions that comprise Fiji (Northern division, Central division, Western division, Eastern division). Inset Map at upper left shows location of Fiji in the Pacific Ocean (white arrow). Source: map downloaded from: https://www.shutterstock.com/fr/image-vector/vector-map-fiji-isolated-illustration-black-379408168

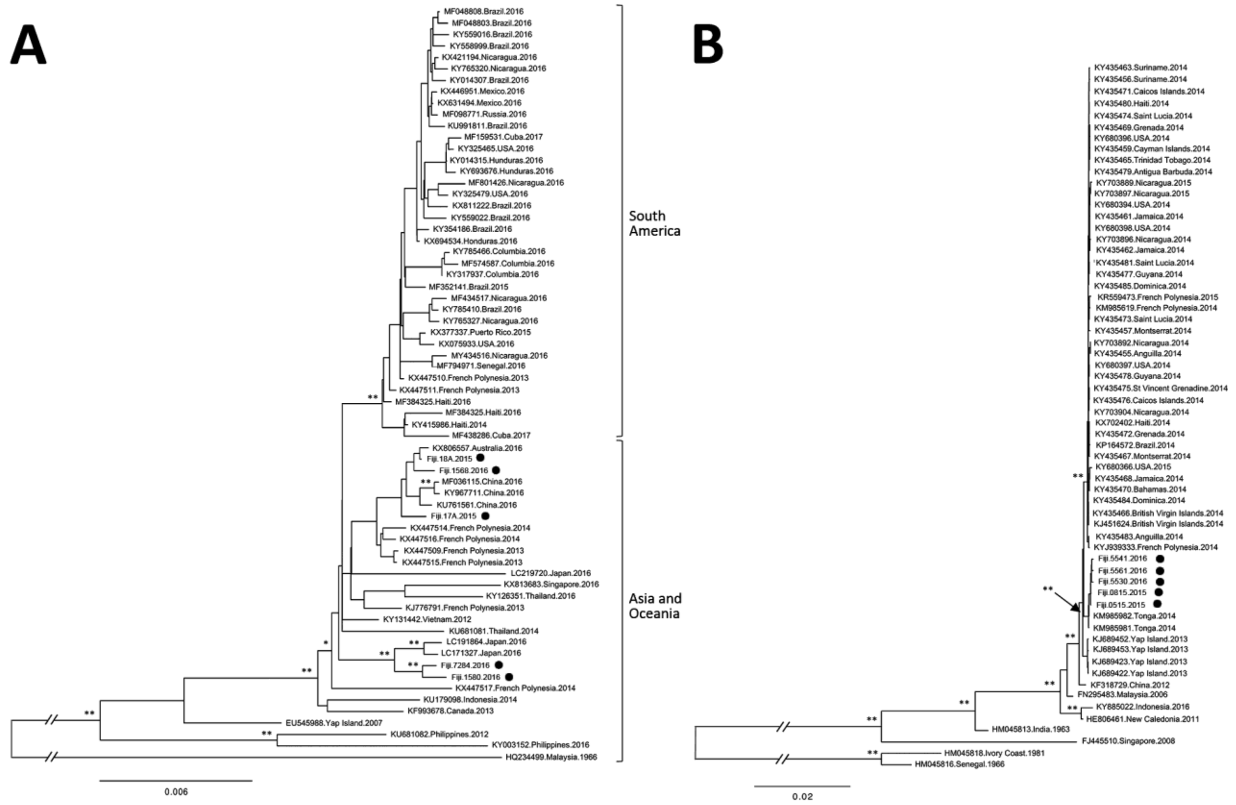

Appendix Figure 2. Phylogenetic relationship between viruses recovered in Fiji and those from other locations. (A) Zika virus E gene and (B) chikungunya virus E1 gene. Analyses were performed using the GTR model of nucleotide substitution with gamma-distributed rate heterogeneity, a lognormal relaxed molecular clock model, and the Bayesian Skyline coalescent tree prior. A mean substitution rate prior of 4x10–4 substitutions per site per year was used for both datasets. The MCMC chains were run for 100 million generations, with 2–4 runs performed for each file. Convergence of the estimates was considered satisfactory when the effective sample size (ESS) calculated in Tracer v1.6.0 (available at: http://tree.bio.ed.ac.uk/software/tracer/) was >200. BEAST log and tree files obtained for a given dataset were combined with LogCombiner v1.8.2, using a burn-in of 10%. Maximum clade credibility trees (MCCT) were summarized using TreeAnnotator v1.8.2, from the BEAST package, keeping the median height over the posterior distribution of trees. MCCT were visualized and edited with FigTree v1.4.2. (available at: http://tree.bio.ed.ac.uk/software/figtree/). Strains collected in Fiji in 2015 and 2016 are tagged with a black circle. Branches with a posterior probability >0.70 or >0.99 are labeled with one (*) or two (**) asterisks, respectively. Each strain is labeled by GenBank accession number/Country/Collection date. Bars indicate the number of nucleotide substitution per site.
